# Multi-omic analysis identifies erythroid cells as the major population in mouse placentas expressing genes for antigen presentation in MHC class II, chemokines, and antibacterial immune response

**DOI:** 10.3389/fimmu.2025.1644983

**Published:** 2025-09-22

**Authors:** Olga Perik-Zavodskaia, Roman Perik-Zavodskii, Saleh Alrhmoun, Kirill Nazarov, Julia Shevchenko, Konstantin Zaitsev, Sergey Sennikov

**Affiliations:** ^1^ Laboratory of Molecular Immunology, Federal State Budgetary Scientific Institution Research Institute of Fundamental and Clinical Immunology, Novosibirsk, Russia; ^2^ Federal State Budgetary Scientific Institution “Siberian Federal Research and Clinical Center of the Federal Medicobiological Agency”, Tomsk, Russia

**Keywords:** pregnancy, fetomaternal tolerance, placenta, spatial transcriptomics, erythroid cells, CD71+ erythroid cells, NanoString, scRNA-seq

## Abstract

**Introduction:**

Pregnancy is a complex process that requires a tightly regulated immune environment to support fetal development and protect against infections. The main barrier between fetus and mother is a specialized organ, the placenta, where the role of specific immune cell populations remains incompletely understood.

**Methods:**

In this work, we used spatial transcriptomics at E12.5 to characterize immune and non-immune cell heterogeneity and spatial organization in the mouse placenta. In addition, we performed murine placental mononuclear cell flow cytometry proteomics, murine placental erythroid cell NanoString bulk transcriptomics, and murine placental erythroid cell LegendPlex secretomics at E12.5 and E19.5 to further analyze the immune landscape in the mouse placenta. We also performed single-cell RNA sequencing of human cord blood erythroid cells for cross-species comparisons.

**Results:**

Our results show that erythroid cells constitute the predominant immunoregulatory population in murine placentas, comprising on average 80% and 40% of the placental mononuclear cells at E12.5 and E19.5, respectively, expressing *Ctss, Cd74, H2-Aa*, and *H2-Ab1* genes involved in antigen presentation via MHC-II, and a PD-L1 checkpoint inhibition molecule gene expression. They also have gene expression of such immunomodulatory molecules as *Tgfb1* and *Tgfb3* cytokines, *Ccl2, Ccl3, Ccl4, Ccl9, Cxcl1, Cxcl12*, and *Mif* chemokines, and antimicrobial protein calprotectin *S100a8, S100a9* genes.

**Discussion:**

These results indicate that erythroid cells may act as potent regulators of immunity in murine placentas due to their vast number and repertoire of immunoregulatory molecules, shaping the immune landscape through diverse immunoregulatory mechanisms.

## 1 Introduction

Pregnancy represents a unique immunological paradox, in which the maternal immune system must simultaneously tolerate the semi-allogeneic fetus while maintaining effective immune defenses against infections (1, 2). A successful pregnancy relies on the precise regulation of immune responses at the maternal-fetal interface in a specialized organ – the placenta (3). The placenta is crucial in maintaining pregnancy by facilitating nutrient and gas exchange between maternal and fetal circulation via cord blood, as well as hormone production and immune regulation (4, 5). A key aspect of placental function is establishing fetomaternal tolerance, which prevents the maternal immune system from rejecting the semi-allogeneic fetus (6, 7). Despite significant progress in understanding placental biology, many aspects of immune regulation at the maternal-fetal interface remain poorly understood. Comparative studies between human and murine placentas provide valuable insights into the evolutionarily conserved and species-specific mechanisms that govern pregnancy success. Murine models also allow for syngeneic pregnancies if mice from the same line are used, thus eliminating the antigen-specific responses occurring in human and cross-mouse line pregnancies. This allows researchers to understand the impact of such responses (5, 8, 9).

The human placenta is hemochorial, characterized by deep trophoblast invasion into the maternal decidua and direct exposure of fetal tissues to maternal blood. Human trophoblast cells, particularly extravillous trophoblasts, play a major role in remodeling spiral arteries and interacting with maternal immune cells (10, 11). In contrast, the murine placenta, while also hemochorial, exhibits a labyrinthine structure with limited trophoblast invasion and retention of maternal endothelial layers (12–14). These structural differences influence the composition and function of immune cells at the maternal-fetal interface, leading to distinct regulatory mechanisms in fetomaternal tolerance (15–17).

Key immune cell populations involved in human fetomaternal tolerance include regulatory T cells (Tregs) (18, 19), M2 macrophages (20, 21), decidual natural killer (dNK) cells (22, 23), and erythroid cells (24, 25). Tregs suppress maternal anti-fetal immune responses through the secretion of IL-10 and TGF-β1, though their accumulation and activity differ between humans and mice (18, 19). M2 macrophages contribute to angiogenesis and immune suppression via VEGF and IL-10 secretion (20, 21), while dNK cells regulate vascular remodeling and trophoblast interactions (22, 23).

In mice, erythroid cells were recently identified as potential regulators of placental immune tolerance in two landmark studies from Elahi and co-authors (26, 27). In their work, they demonstrated that erythroid cells constitutively display the checkpoint ligand PD-L1 and express arginase-2, endowing them with dual tolerogenic machinery that curtails effector T-cell activation and cytokine production while preserving tissue homeostasis at the fetomaternal interface. Functionally, this program enforced an effector T-cell-poor milieu – a practical T-cell desert within murine placental tissues, and, crucially, biases the immune landscape toward regulatory T-cell differentiation and maintenance (26). Extending the scope of this erythroid-centered tolerance beyond the placenta, this collective of authors also showed that erythroid cells during pregnancy and in the neonatal period foster symbiotic intestinal microbial communities, revealing a coordinated, system-level immunoregulatory axis that links placental tolerance with postnatal mucosal immune education and microbial stewardship (27). They also showed that the depletion of erythroid cells using anti-CD71 antibodies results in pregnancy failure and even suggested that “erythroid cells are essential in fetomaternal tolerance” (26, 27), which may be due to the elimination of RBCs and their precursors in both the placenta and peripheral tissues, since CD71 is an important marker of maturation of almost the entire erythron – BFU-E (burst-forming unit-erythroid), CFU-E (colony-forming unit-erythroid), proerythroblast, basophilic erythroblast, polychromatophilic erythroblast, orthochromatophilic erythroblast, and early reticulocytes (28). It has also been shown that human and mouse erythroid cells are capable of cytokine gene expression and secretion (29, 30).

Given the importance of placental immune regulation in pregnancy outcomes, continued research using modern transcriptomic approaches in both humans and mice is essential. Understanding the cellular and molecular mechanisms governing fetomaternal tolerance will not only enhance our knowledge of normal pregnancy physiology but also aid in developing therapeutic strategies for pregnancy complications such as preeclampsia, recurrent miscarriage, and fetal growth restriction. To further dissect the complexity of placental immune regulation, we decided to use spatial transcriptomics to study the characterization of immune and non-immune cell heterogeneity and spatial organization within the murine placenta at E12.5, as well as perform murine placental immune cell population analysis via flow cytometry proteomics, NanoString bulk transcriptomics, and LegendPlex secretomics. We also performed scRNA-seq of human placenta-derived cord blood erythroid cells for the interspecies comparative study.

## 2 Materials and methods

### 2.1 Acquisition of murine placentas

#### 2.1.1 Murine pregnancy modeling

We modeled semi-allogeneic pregnancy ♀CBA × ♂C57Bl6 (*n* = 19) and syngeneic pregnancy ♀CBA × ♂CBA (*n* = 19) to obtain mouse placentas. One male and one female of the appropriate genetic line were co-housed per cage. Mice were obtained from the vivarium of the Institute of Cytology and Genetics (Novosibirsk, Russia). Mice were kept under normal vivarium conditions with access to water and food ad libitum, in a natural day and night cycle. The onset of pregnancy was determined by the appearance of a vaginal plug in females. Euthanasia and collection of fetuses with placentas were performed on the 12th–13th day after mating of mice (E12.5, *n* = 11 for each type of pregnancy) and the 19th–20th day after mating of mice (E19.5, *n* = 8 for each type of pregnancy). Pregnant mice were euthanized by cervical dislocation.

After euthanizing pregnant mice, we opened the abdominal cavity under aseptic conditions and carefully removed the fetuses along with the placentas. We then transferred some of the fetoplacental complexes to a cryomold (*n* = 3 for each type of pregnancy at E12.5) and used others to extract placental mononuclear cells (*n* = 8 for each type of pregnancy at both E12.5 and E19.5).

#### 2.1.2 Cryosectioning of murine placentas

We froze fetuses with placentas in OCT tissue freezing solution at −20°C, sectioned them on a cryostat at 10 μm, and transferred the resulting sections to Poly-Prep Slides (P0425 - 72EA, Sigma-Aldrich) for fresh frozen sectioning. We then immediately used the obtained sections to prepare 10X Genomics Visium spatial transcriptomics libraries on a CytAssist instrument. Since the CytAssist instrument can only process two samples simultaneously, we always processed one semi-allogeneic and one syngeneic fetoplacental section together for batch effect removal.

#### 2.1.3 Isolation of mouse placental MNCs

To analyze placental cell populations, we transferred fetuses with placentas to a sterile Petri dish, where we separated the fetus from the placenta. We placed the isolated placentas in sterile glass vials containing cold RPMI - 1640 medium with antibiotics. Placentas from each mouse were pooled. We isolated placental cells by homogenizing whole placentas in a glass homogenizer in 3 – 5 mL of PBS. After washing the cell suspension in 5 – 10 mL of PBS, we centrifuged the placental cells in a Ficoll-Urografin density gradient (density = 1.119 g/ml) for 30 min at RCF 322 and washed twice in PBS.

#### 2.1.4 Magnetic separation of placental Ter-119^+^ erythroid cells

We performed magnetic separation of mouse placental mononuclear cells using biotinylated anti-Ter-119 antibodies (#116203, Biolegend, San Diego, USA) and streptavidin-coupled magnetic beads (#480015, Biolegend, San Diego, USA) for all experiments except spatial transcriptomics and flow cytometry analysis.

#### 2.1.5 Viability staining of placental Ter-119^+^ erythroid cells

We measured the placental Ter-119^+^ erythroid cell viability on a Countess 3 Automated Cell Counter (Thermo Fisher Scientific, Waltham, USA) according to the manufacturer’s protocols using Trypan Blue. Trypan Blue staining showed >98% viability for the sorted erythroid cells.

### 2.2 Spatial transcriptomic analysis of murine placentas

#### 2.2.1 Preparation of 10X genomics Visium spatial transcriptomics libraries

We performed library preparation of the murine placentas according to the manufacturer’s instructions for the Visium CytAssist Spatial Gene Expression for Fresh Frozen (*n* = 3 for each type of pregnancy at E12.5, see **Supplementary Methods** for the detailed procedure).

#### 2.2.2 Single-cell level deconvolution of 10X Visium data via CoexpressDeconvolve

We used CoexpressDeconvolve (31) (https://github.com/Perik-Zavodskii/CoexpressDeconvolve, accessed on 21.05.2025) to perform single-cell state deep deconvolution of 10X Visium data. In brief, CoexpressDeconvolve used a gene expression matrix in the market exchange format to calculate the Gene x Gene distance matrix by binarizing gene UMI counts, computing Pearson correlation coefficients, and converting them into distance values by subtracting them from 1. It then identified distance-based gene clusters using a hierarchical clustering algorithm with a resolution of 1.5. The resolution of 1.5 was chosen because it clearly separated all major cell types in mouse fetoplacental complexes. For each distance-based gene cluster, the pipeline fitted a Negative Binomial distribution and applied the 85th percentile distribution threshold to identify clusters within spatial spots. To estimate cell counts, the minimum UMI sum for each distance-based cluster was computed, and the UMI sum of each distance cluster per spot was divided by the distance cluster UMI sum minimum. Using this information, the pipeline deconvolved spatial transcriptomic spots into distance clusters based on their gene expression profiles and further deconvolved these distance clusters into putative single-cells. Finally, it randomly allocated the putative cells within their original spatial spot locations and generated gene expression, cell location, and cell cluster output files.

#### 2.2.3 Spatial transcriptomic analysis of the single-cell-deconvolved murine placentas in Seurat

We imported the gene expression, cell location, and cell cluster output files from CoexpressDeconvolve into Seurat (32), performed log-normalization, performed cluster identification via GSEApy (33) using the distance cluster genes, used *SpatialDimPlot()* and *SpatialFeaturePlot()* to create spatial expression plots, found top genes for each cluster, and created a dot plot of the genes via the *DotPlot()* function.

### 2.3 Flow cytometry analysis of the placental mononuclear cells

#### 2.3.1 Cell staining and data acquisition via flow cytometry

We washed 1 – 2 × 10^6^ placental mononuclear cells (*n* = 4 for each type of pregnancy at both E12.5 and E19.5) in PBS supplemented with 0.09% NaN_3_ and stained them with antibodies according to the manufacturer’s protocols. For flow cytometry proteomic analysis, we used the following antibodies: FITC anti-mouse CD14 (Cat # 123308), PE/Cyanine7 anti-mouse CD8a (Cat # 100722), APC anti-mouse CD71 (Cat # 113820), APC/Cyanine7 anti-mouse CD45 (Cat # 103116), Pacific Blue anti-mouse TER - 119/erythroid cells (Cat # 116232), and Brilliant Violet 570 anti-mouse CD4 (Cat # 100542) (BioLegend, USA). After incubating the cells in the dark for 30 – 40 minutes, we washed them with 0.5 ml PBS supplemented with 0.09% NaN_3_. Flow cytometry was performed on an Attune NxT flow cytometer (Thermo Fisher Scientific, USA).

#### 2.3.2 Flow cytometry data analysis

To analyze the cellular composition of the placenta, we first gated cells and removed doublets, and collected the cell population percentage data of CD4 T-cells, CD8 T-cells, Monocytes, and erythroid nucleated cells (CD71^+^Ter-119^+^) and nucleated reticulocytes (CD71^-^Ter-119^+^) among placental mononuclear cells. We performed statistical analysis using two-way ANOVA with Tukey’s multiple testing correction in GraphPad Prism 10.2.3, considering q-values < 0.005 as statistically significant. We also used GraphPad Prism 10.2.3 to generate the dot plot of the cell cluster percentages.

### 2.4 NanoString gene expression analysis of placental Ter-119^+^ erythroid cells

#### 2.4.1 Isolation of total RNA from mouse placental Ter-119^+^ erythroid cells

Using the Total RNA Purification Plus Kit, we isolated total RNA from 500,000 Ter-119 magnetically separated placental erythroid cells (Norgen Biotek, Canada). We measured the total RNA concentration for each sample using a Qubit 4 fluorometer (Thermo Fisher Scientific, USA). The RNA was then frozen at −80 °C until gene expression analysis.

#### 2.4.2 Immune transcriptome profiling of mouse placental Ter-119^+^ erythroid cells using the NanoString Sprint

We conducted immune transcriptome expression profiling of placental erythroid cells using the NanoString nCounter Sprint Profiler Assay System. We used 200 ng of total RNA from each placental erythroid cell sample and the nCounter Mouse Immunology v1 panel (561 immune-related genes, 15 housekeeping genes, 6 positive and 8 negative controls). We subjected the total RNA samples (*n* = 3 for each type of pregnancy at both E12.5 and E19.5) to a 20-hour hybridization reaction at 65 °C, where we combined 5 – 19 μl of total RNA with 3 μl of reporter probes, 0 – 14 μl of nuclease-free water, 12 μl of hybridization buffer, and 5 μl of capture probes (total reaction volume = 36 μl). After probe hybridization with the targets of interest, we determined the number of target molecules using a NanoString nCounter Sprint.

We performed normalization and quality control in the nSolver 4 software, taking into account the added synthetic positive controls and the expression of housekeeping genes (*Alas1, Gusb, Oaz1, Polr2a, Ppia, Rpl19*, and *Sdha*) included in the panel. We filtered the gene expression matrix by removing non-expressed genes. For this, we subjected the expression data to log2 transformation, and we defined the expression threshold as the median of the transformed normalized values of the number of determined probe copies. We removed genes that were below the expression threshold in all samples.

#### 2.4.3 Gene expression analysis of mouse placental Ter-119^+^ erythroid cells

To investigate the immunoregulatory properties of placental erythroid cells, we analyzed the gene expression profiles of placental erythroid cells, as well as M1 and M2 macrophages. We downloaded raw probe count data for M1 and M2 macrophages from the Gene Expression Omnibus database (GSE120254). We utilized PyDESeq2 (34) for gene expression analysis. We normalized raw probe counts using the median-of-ratios method and performed differential gene expression. We considered genes with *q*-value < 0.001 and log2(FoldChange) > 1.0 or log2(FoldChange) < −1.0 as differentially expressed. To visualize the gene expression, we generated heat maps via bioinfokit (35). We performed Gene Set Enrichment Analyses of the genes expressed by the placental erythroid cells via GSEApy (33).

### 2.5 LegendPlex secretome profiling of murine placental Ter-119^+^ erythroid cells

#### 2.5.1 Cultivation of Ter-119^+^ erythroid cells from mouse placenta

As performed previously (36–38), we cultured magnetically separated Ter-119^+^ placental erythroid cells in serum-free X-VIVO 10 media supplemented with insulin-transferrin for 24 h at a seeding density of 200,000 cells per 200 μl of medium (*n* = 6 for each type of pregnancy at both E12.5 and E19.5). After 24 h of culture, we separated the conditioned medium from the cells by centrifugation at 1500 rpm for 10 min. We transferred the conditioned medium to new 0.2 μl tubes and froze it at −80°C until cytokine quantification.

#### 2.5.2 Analysis of the secretome of Ter-119^+^ erythroid cells from the mouse placenta using LegendPlex

We analyzed cytokine and chemokine levels in conditioned media obtained from Ter-119^+^ erythroid cells of mouse placentas (*n* = 4 for each type of pregnancy at both E12.5 and E19.5) using the LEGENDplex™ multiplex immunoassay. For this purpose, we used LEGENDplex™ Mouse HSC Panel (13-plex) (BioLegend, USA). We performed the assay according to the manufacturer’s recommendations. In brief, we incubated conditioned media samples and standards with beads in 96-well plates with capture beads for 2 hours with gentle shaking. Then, we added biotinylated detection antibodies and incubated the mixture for 1 hour with gentle shaking. After that, we added streptavidin-conjugated fluorochrome-bound antibodies to the wells, performed a final 30-minute incubation with gentle shaking, and then washed the samples to remove unbound components. We performed the fluorescent signal readout on an Attune NxT flow cytometer (Thermo Fisher Scientific, USA). We then processed the data using LEGENDplex™ Data Analysis Software (BioLegend, USA).

To analyze differential cytokine production, we performed multiple t-tests with False Discovery Rate multiple testing correction in BulkOmicsTools (39). We considered Log2(FoldChange) > 2.0 or log2(FoldChange) < −2.0 and *q*-values < 0.01 as statistically significant.

### 2.6 Bacterial growth inhibition assay

To assess the antibacterial activity of erythroid cell-secreted factors during murine pregnancy, we used the collected conditioned media from placental erythroid cells at both E12.5 and E19.5 in both types of pregnancy (*n* = 6). We performed bacterial growth inhibition assays using the *E. coli* NebStable strain (New England Biolabs, USA). Bacteria were cultured overnight in LB broth, diluted to an initial OD600 of 0.05, and mixed with erythroid cell-conditioned media (LBB 4:1 Media). We used LB broth with bacteria as the positive control, LBB with bacteria supplemented with 50 μg/mL kanamycin as the negative control, and LBB without bacteria as a blank. We performed the assay in doublets. Absorbance at 595 nm was measured using the Varioskan Lux plate reader (Thermo Fisher Scientific, Waltham, Massachusetts, USA) every 15 minutes for 18 hours. The plate was maintained at 30°C and continuously shaken at 60 rpm throughout the experiment.

### 2.7 Analysis of single human placenta-derived cord blood CD235a^+^ erythroid cells

#### 2.7.1 Magnetic separation of human cord blood CD235a^+^ erythroid cells

We obtained cord blood samples stored in 10% DMSO and 90% FBS from “Bank Stvolovih Kletok” LLC. As performed in our previous human erythroid cell study (40), we thawed cord blood samples (*n* = 3) stored in liquid nitrogen in a 37°C water bath and washed them with 6 ml of a mixture containing 5 ml of complete RPMI 1640 cell culture medium and 1 ml of FBS. We isolated cord blood mononuclear cells by density gradient centrifugation (Ficoll-Paque 1.077 g/ml) at 266 RCF for 30 min. We performed magnetic sorting of cord blood mononuclear cells using anti-CD235a MicroBeads (130 - 050-501, Miltenyi Biotec, Germany) according to the manufacturer’s protocols.

#### 2.7.2 BD Rhapsody library preparation

As performed in our previous human erythroid cell study (40), we incubated magnetically separated erythroid cells with Sample Tag molecular barcoding antibodies for the BD Rhapsody System for 20 minutes at room temperature and then washed them three times with cold sample buffer (BD Biosciences, USA). After the washing cycles, we counted the cord blood erythroid cells using Calcein cell staining and an Attune NxT flow cytometer. We pooled equal numbers of cells, resuspended them in a cold sample buffer to a final concentration of 20 cells/μl, and loaded them onto a BD Rhapsody cartridge. We assessed the quality of cell loading using an InCell Analyzer 6000.

We performed single-cell capture and cDNA library preparation using the BD Rhapsody Express Single-Cell Analysis System (BD Biosciences, USA) according to the manufacturer’s instructions. Briefly, we amplified cDNA in 10 PCR cycles using the Immune Response primer panel (633750, BD Biosciences, USA), which contains 399 primer pairs targeting 397 different genes. We purified the resulting PCR1 products using AMPure XP magnetic beads (Beckman Coulter, USA) and separated the Immune Response panel library amplicons from the Sample Tag library based on amplicon size.

Next, we amplified the purified PCR1 Immune Response panel library products and the Sample Tag PCR1 library products in 10 semi-nested PCR cycles for each library. We purified the resulting PCR2 products by two-way selection using AMPure XP beads. We estimated the concentration of the final libraries using Qubit 4.0 (High-Sensitivity dsDNA Kit, Thermo Fisher Scientific, USA). We normalized the final PCR products to 2.5 ng/µl for the Immune Response panel library and 1.0 ng/µl for the Sample Tag library and performed a final round of amplification (8 PCR cycles for both the Immune Response panel library and for the Sample Tag library) using index primers for the Illumina sequencer to prepare the final libraries. We performed library QC on a Qsep 1 capillary electrophoresis apparatus (BiOptic Inc., Taiwan).

We pooled the final libraries at an Immune Response panel/Sample Tag library ratio of ~94%/6%, aiming for approximately 10,000 (Immune Response panel) and 600 (Sample Tag) reads per cell, to achieve a final concentration of 5 nM. We sequenced the pooled libraries on a NextSeq 550 sequencer (Illumina, USA) (130 million paired reads, R1 = 75, R2 = 75).

#### 2.7.3 Analysis of raw sequencing data

We processed the raw FASTQ files resulting from sequencing using the BD Rhapsody v1.9 pipeline (BD Biosciences). The pipeline first removed low-quality read pairs based on their read length, average base quality score, and highest single-nucleotide frequency; then analyzed the remaining high-quality R1 reads to identify Cell Label and UMI sequences; and aligned the remaining high-quality R2 reads to the mRNA reference panel (Immune Response panel) sequences using Bowtie2 (41). The pipeline then collapsed reads with a common Cell Label, UMI, and gene into unique transcript molecules. The pipeline adjusted the resulting counts using error correction algorithms, namely recursive substitution error correction and distribution-based error correction, to adjust for sequencing and PCR errors. The pipeline then separated cells from noise using a second derivative analysis. The pipeline then used Sample Tag information to demultiplex samples and remove multiplets. The pipeline detected 9.615 erythroid cells from human cord blood.

#### 2.7.4 Immune transcriptome expression analysis

We subjected the obtained gene expression matrices of the cord blood erythroid cells to our established erythroid cell analysis Seurat pipeline (32, 42, 43), we performed quality control and SCTransform data normalization, conducted PCA (principal component analysis) dimensionality reduction on the normalized data, performed Harmony batch correction and integration, used 20 Harmony-corrected principal components for UMAP dimensionality reduction, and found single-cell neighbors and clusters.

We then identified erythroid cell clusters corresponding to subsequent stages of erythroid cell differentiation. We prepared SCT markers for differential gene expression testing using the *PrepSCTFindMarkers()* function and performed inter-cluster differential gene expression using the Wilcoxon signed-rank test. We set biological and statistical significance criteria as log2(FoldChange) > 1.0 or log2(FoldChange) < −1.0 and q-value < 0.005 using the *FindMarkers()* function. We identified clusters using the differentially expressed *ALAS2, CD36*, and *ITGA4* genes. The expression of *ALAS2, SCL25A37*, and *SCNA* genes gradually increased from the proerythroblast stage onward, while the expression of *CD36* and *ITGA4* genes gradually decreased from the proerythroblast stage. We found that *ARG1* ^+^ erythroid cells had a unique expression of the *ARG1* gene. Additionally, we detected a small subpopulation of erythroid cells – *DEFA3*+ erythroid cells that had *DEFA3* gene expression. Finally, we generated a dot plot of differentially expressed genes using *DotPlot* in Seurat (32).

To compare the gene expression between the human cord blood and murine placental erythroid cells, we aggregated UMI counts per sample for each scRNA-seq sample, converted mouse gene names to human, normalized raw probe counts of both NanoString and aggregated scRNA-seq samples using the median-of-ratios method, performed log2-based data transformation, and performed Z-score data standardization. We visualized the gene expression via bioinfokit using a heatmap (35). We also performed R^2^ calculation to visualize the gene expression differences between the human cord blood and murine placental erythroid cells via the Pandas *corr* function, and visualized the gene expression via Matplotlib.

## 3 Results

### 3.1 Erythroid lineage cells are the most common immunoregulatory cells in murine placentas

**Figure 1:**
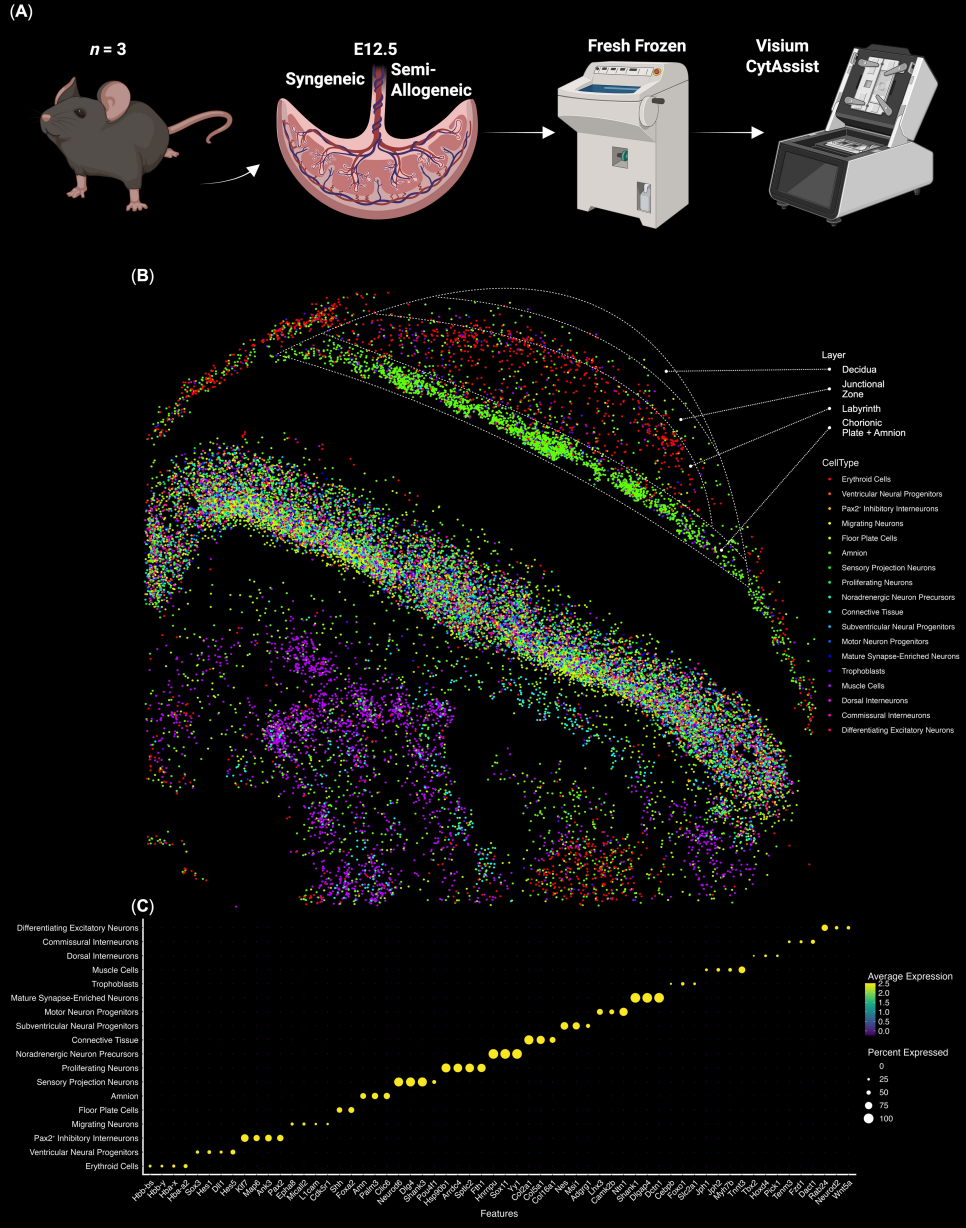
Spatial transcriptomic analysis of the E12.5 murine fetoplacental complex. **(A)** Overview of the experiment, **(B)** The Spatial plot of the representative E12.5 murine fetoplacental complex (27.313 single cells), erythroid cells are colored red; **(C)** Dot plot of the E12.5 murine fetoplacental complex cluster marker genes, dot size indicates the percentage of cells positive for a marker. Dashed lines indicate placental layers; cells outside the explicitly outlined placental layers belong to fetal and perifetal tissues.

To study the immune populations in mouse placentas, we performed a spatial transcriptomics analysis of murine placentas at E12.5 from both semi-allogeneic and syngeneic pregnancies (*n* = 3). First, we performed the single-cell level of spatial data deconvolution via CoexpressDeconvolve and single-cell cluster identification. We observed that the erythroid lineage cells were the only detectable immunoregulatory cell population among murine placentas in both syngeneic and semi-allogeneic pregnancies. We also observed that erythroid cells formed distinct niches, were present in all layers of the placenta: decidua, junctional zone, and labyrinth, and expressed both fetal (*Hba-x*/*Hbb-y*) and normal (*Hba-a2*/*Hbb-bs*) hemoglobin, which suggests the presence of both fetal and maternal erythroid cells (**Figure 1**, See **Supplementary Figure 1**).

**Figure 2:**
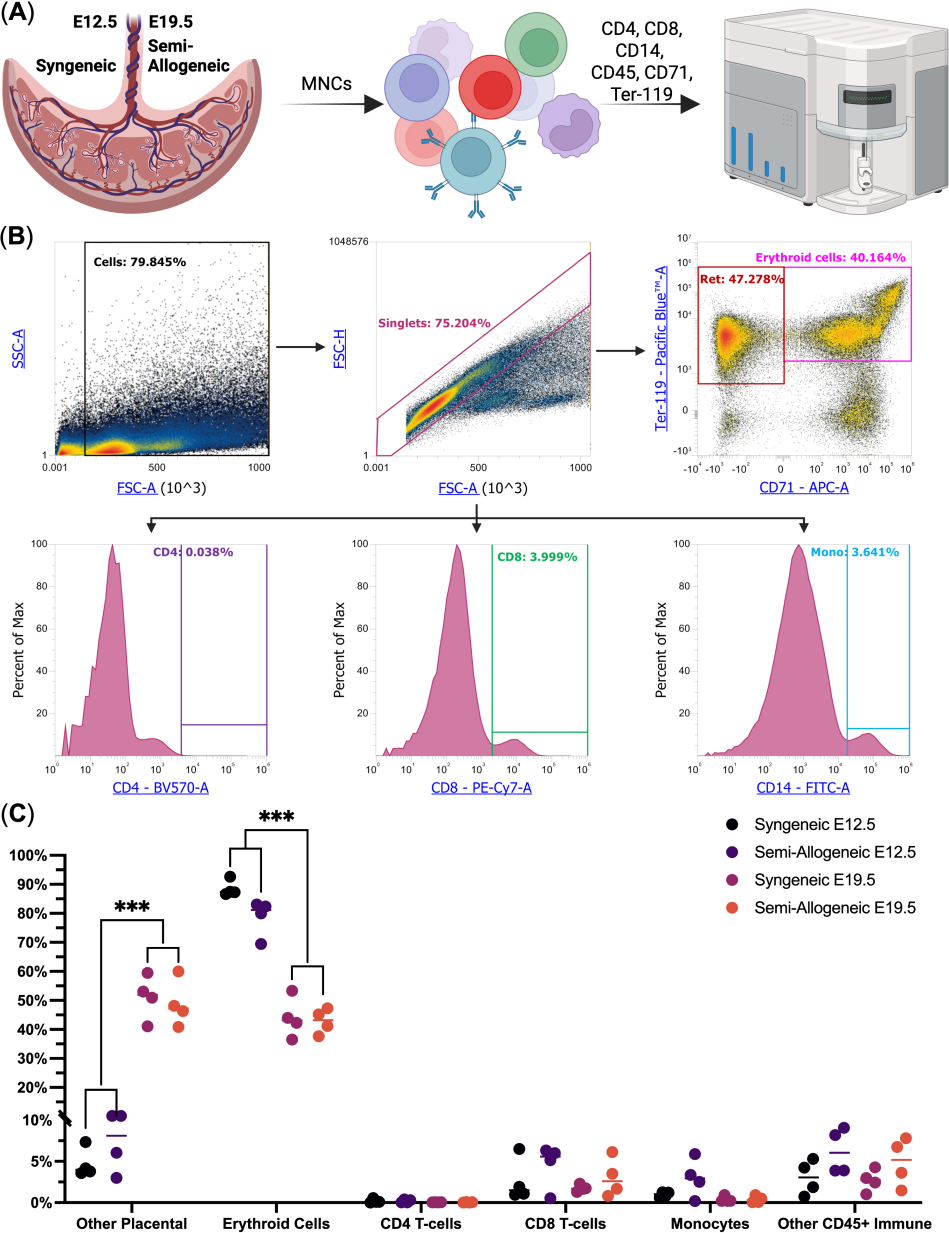
Flow cytometry analysis of the murine placental mononuclear cells at E12.5 and E19.5 from syngeneic and semi-allogeneic pregnancies (*n* = 4). **(A)** Overview of the experiment; **(B)** Flow Cytometry gating scheme, Ret. – nuclei-containing reticulocytes, CD4 – CD4^+^ T-cells, CD8 – CD8^+^ T-cells, Mono – CD14^+^ Monocytes; **(C)** Dot plot of the studied cell populations, *** - *q*-values < 0.005.

We then used flow cytometry to study the immune cell population in murine placentas. We observed that Ter-119^+^ erythroid lineage cells (Ter-119^+^ nuclei-containing reticulocytes and nucleated erythroid cells) were indeed the dominant cell population among placental mononuclear cells, thus confirming our spatial transcriptomics data at E12.5 – erythroid lineage cells comprised, on average, 88.5 ± 2.0% of placental mononuclear cells in syngeneic pregnancy and 78.5 ± 6.3% in semi-allogeneic pregnancy. We also observed that E19.5 marked a significant decrease in the percentage of placental erythroid cells – they comprised 43.1 ± 4.8% of the placental mononuclear cells, regardless of the type of pregnancy (a 1.9 – 2.1-fold decline). Other immune cell types comprised 2.1 ± 2.4% of the placental mononuclear cells, thus validating our spatial transcriptomic findings (**Figure 2**, See **Supplementary Table 1**).

### 3.2 Murine placental erythroid cells express genes involved in immunosuppression, MHC class II antigen presentation, immune cell chemotaxis, and antibacterial immunity

**Figure 3:**
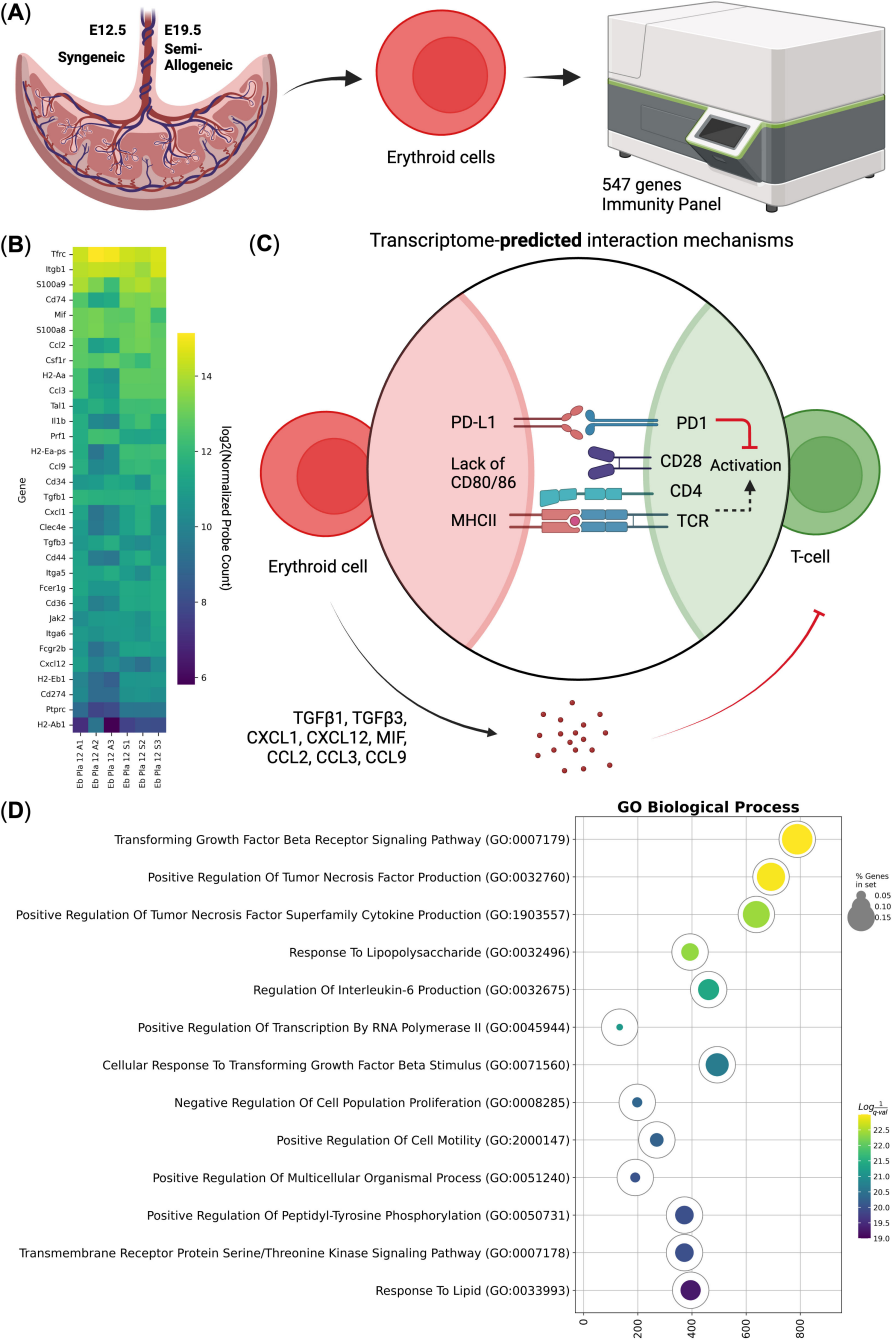
Immunotranscriptomic analysis of murine placental erythroid cells (Eb) from syngeneic (S) and semi-allogeneic (A) pregnancies. **(A)** Overview of the experiment, **(B)** Heat map of the immunoregulatory log2-transformed normalized probe counts of the genes expressed by the murine placental erythroid cells at E12.5 from both syngeneic and semi-allogeneic pregnancies, **(C)** Visualization of the predicted interaction with the CD4 T-helper cells by murine placental E12.5 erythroid cells, **(D)** Gene Ontology Biological Process overrepresentation analysis of the genes with the detected expression in murine placental E12.5 erythroid Cells. The yellow color corresponds to the lowest *q*-value, the purple color corresponds to the highest *q*-value, and the dot size reflects the percentage of genes in the analysis from the full set of genes in the Gene Ontology Biological Process database.

**Figure 4:**
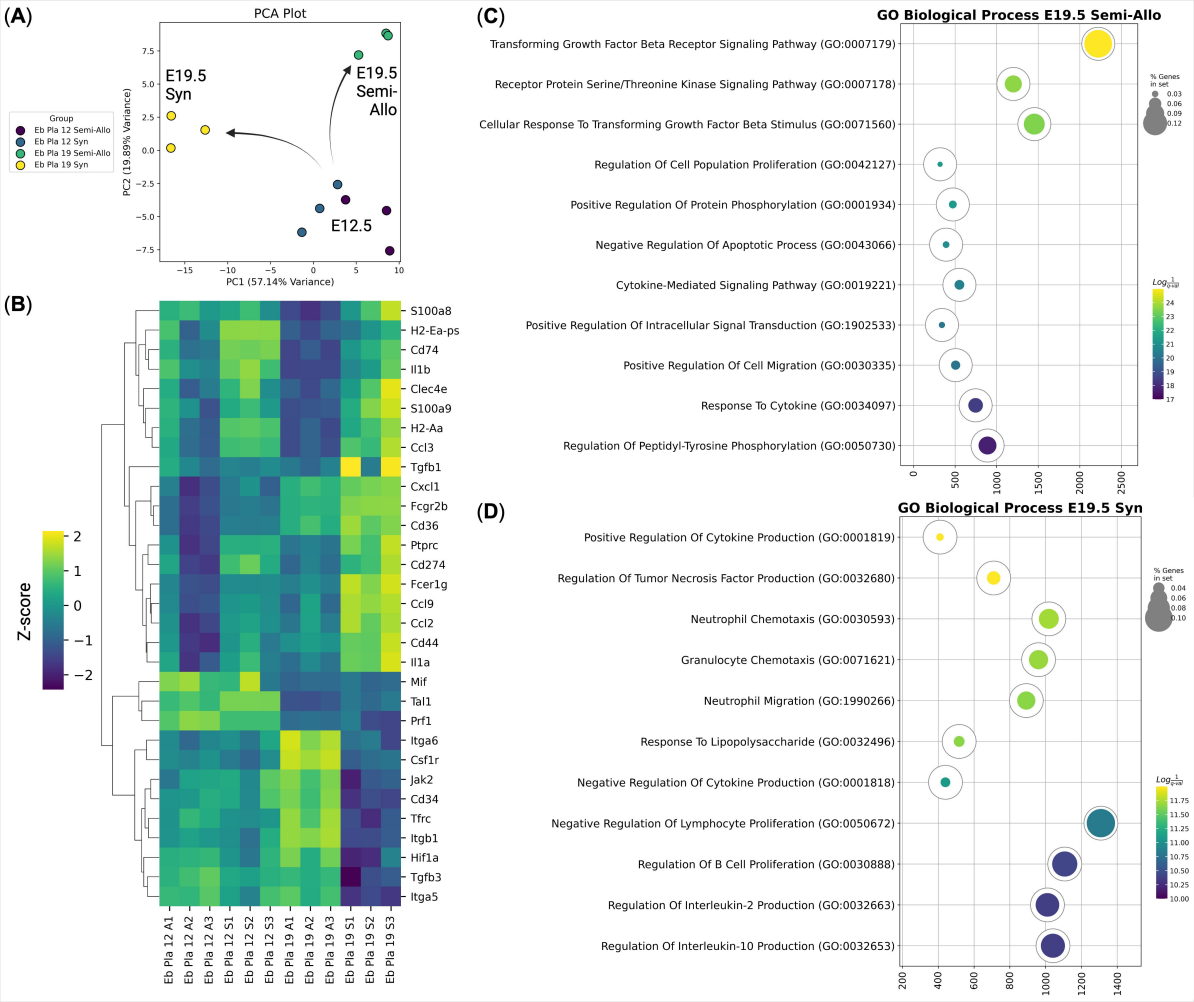
Immunotranscriptomic analysis of murine placental erythroid cells (Eb) from syngeneic (S) and semi-allogeneic (A) pregnancies at E12.5 and E19.5 (*n* = 3). **(A)** PCA plot of the samples, arrows show the transcriptional divergence of the murine placental erythroid cells at E19.5; **(B)** Heat map of the immunoregulatory genes expressed by the murine placental erythroid cells at E12.5 and E19.5 from syngeneic and semi-allogeneic pregnancies, gene expression values are Z-score-transformed; **(C)** Gene Ontology Biological Process overrepresentation analysis of the genes with the up-regulated expression in murine placental semi-allogeneic E19.5 erythroid cells. The yellow color corresponds to the lowest *q*-value, the purple color corresponds to the highest *q*-value, and the dot size reflects the percentage of genes in the analysis from the full set of genes in the Gene Ontology Biological Process database, **(D)** Gene Ontology Biological Process overrepresentation analysis of the genes with the up-regulated expression in murine placental syngeneic E19.5 erythroid cells. The yellow color corresponds to the lowest *q*-value, the purple color corresponds to the highest *q*-value, and the dot size reflects the percentage of genes in the analysis from the full set of genes in the Gene Ontology Biological Process database.

As Ter-119^+^ erythroid cells were indeed overrepresented among the murine placental mononuclear cells at E12.5 and E19.5 in both syngeneic and semi-allogeneic pregnancies, we next performed positive magnetic separation of placental Ter-119^+^ erythroid cells followed by their immunotranscriptomic analysis via NanoString (**Figures 3**, **4**). Our gene expression analysis (**Figure 3A**) revealed that murine placental erythroid cells at E12.5 had identical transcriptomic profiles in both types of pregnancy and that they expressed a plethora of immunoregulatory genes, including PD-L1 (*Cd274* gene), MHC class II antigen presentation genes, and cytokine and chemokine genes such as *Tgfb1, Tgfb3, Cxcl1, Cxcl12, Mif, Ccl2, Ccl3, Ccl4*, and *Ccl9* (**Figures 3B, C**).

We then performed Gene Ontology (GO) enrichment analysis of the genes expressed in the E12.5 murine placental erythroid cells and identified several significantly enriched pathways, including TGF-β receptor signaling and response to lipopolysaccharide, which may indicate a dual role for murine placental erythroid cells as both antimicrobial protectors and immune tolerance inducers (**Figure 3D**).

To compare the transcriptional profiles of placental erythroid cells at different gestational stages, we compared murine placental erythroid cells at E12.5 and E19.5 from both syngeneic and semi-allogeneic pregnancies (**Figure 4**). Principal component analysis (PCA) of gene expression profiles revealed a marked transcriptional divergence between E12.5 and E19.5 erythroid cells. Notably, E19.5 placental erythroid cells clustered according to pregnancy type, indicating that these cells may acquire distinct transcriptional profiles (and potentially distinct immunoregulatory roles) depending on the maternal-fetal immune context (**Figure 4A**).

Differential gene expression analysis of the murine placental erythroid cells (**Figure 4B**) revealed that E19.5 syngeneic pregnancy erythroid cells expressed genes associated with inflammatory and antimicrobial responses, including *Il1a, Il1b, Clec4e, Ccl2, Ccl3, Ccl9, S100a8*, and *S100a9*. In contrast, genes upregulated in E19.5 erythroid cells from semi-allogeneic pregnancies involved in regulation via the TGF-beta signaling pathway (**Figures 4C, D**, **Supplementary Figures 2**,**3**,**4**).

To explore the overall immune regulation polarization status of murine placental erythroid cells, we analyzed gene expression profiles from M1 and M2 macrophages (GSE120254) alongside erythroid cells from E12.5 and E19.5 placentas. Compared with both M1 and M2 macrophages, murine placental erythroid cells at both E12.5 and E19.5 displayed a significantly higher expression of such inflammatory and antibacterial markers as *Il1a, Il1b, S100a8*, and *S100a9*. Murine placental erythroid cells also showed higher expression of the *Cd274* gene (PD-L1) compared with M1 macrophages and lower *Tgfb1* gene expression compared to both M1 and M2 macrophages (**Supplementary Figure 5**).

We also assessed the *in vitro* antibacterial activity of murine placental erythroid cell conditioned media, as they had high *S100a8* and *S100a9* (antimicrobial peptide calprotectin) (44–46) gene expression. We observed that murine placental erythroid cell conditioned media completely inhibited the growth of *E. coli*, akin to the negative kanamycin control (**Supplementary Figure 6**).

### 3.3 Murine semi-allogeneic pregnancy placental erythroid cells secrete significantly more TGF-β1 compared with their syngeneic counterparts

**Figure 5:**
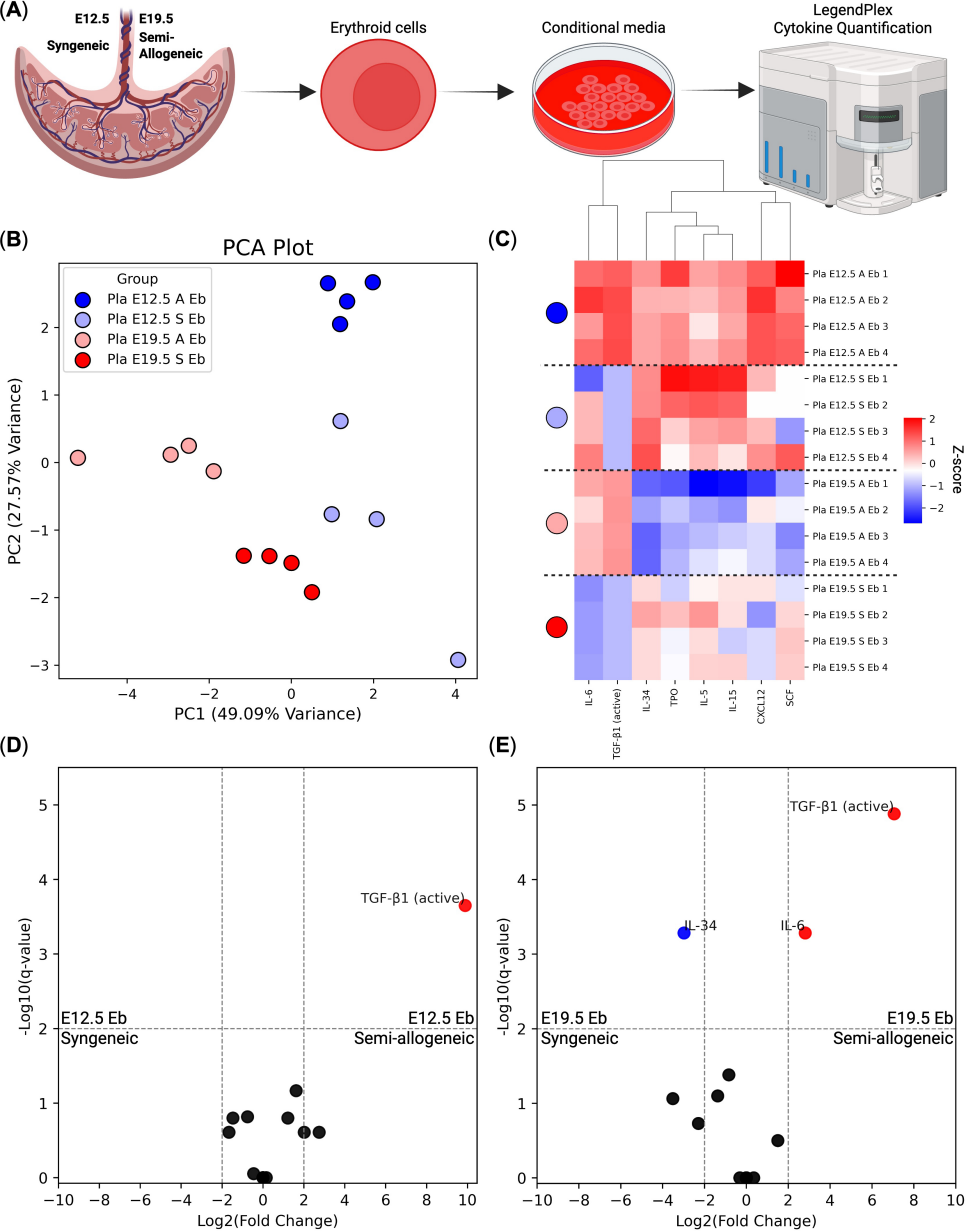
LegendPlex cytokine profile of placental (Pla) Ter-119^+^ erythroid cell (Eb)-derived conditioned media at E12.5 and E19.5 in both syngeneic (S) and semi-allogeneic pregnancies (A) (*n* = 4). **(A)** Overview of the experiment, **(B)** Principal component analysis (PCA) of cytokine secretion profiles, **(C)** Heat map of the Z-score transformed cytokine concentration values, **(D)** Volcano plot comparing cytokine levels between E12.5 erythroid cells from syngeneic and semi-allogeneic pregnancies, **(E)** Volcano plot comparing cytokine levels between E19.5 erythroid cells from syngeneic and semi-allogeneic pregnancies. We considered log2(Fold Change) > |2| and *q*-values < 0.01 significant.

To investigate the immunoregulatory properties of placental erythroid cells, we collected conditioned media from Ter-119^+^ erythroid cells and analyzed their secretome using the LegendPlex cytokine quantification platform (**Figure 5A**, see **Supplementary Table 2**). Principal component analysis revealed distinct clustering of erythroid-derived media according to gestational age and maternal-fetal context, indicating substantial variation in secreted cytokine profiles between groups (**Figure 5B**).

Differential cytokine production analysis identified that TGF-β1 was significantly more produced in semi-allogeneic pregnancy erythroid cell conditioned media compared with syngeneic pregnancy erythroid cell conditioned media (**Figures 5C–E**), suggesting enhanced immunomodulatory activity might be required by murine placental erythroid cells to alleviate the fetomaternal antigenic conflict in semi-allogeneic pregnancy at both E12.5 and E19.5.

### 3.4 Human cord blood erythroid cells express immunosuppressive and antibacterial genes in accord with the murine placental erythroid cells

**Figure 6:**
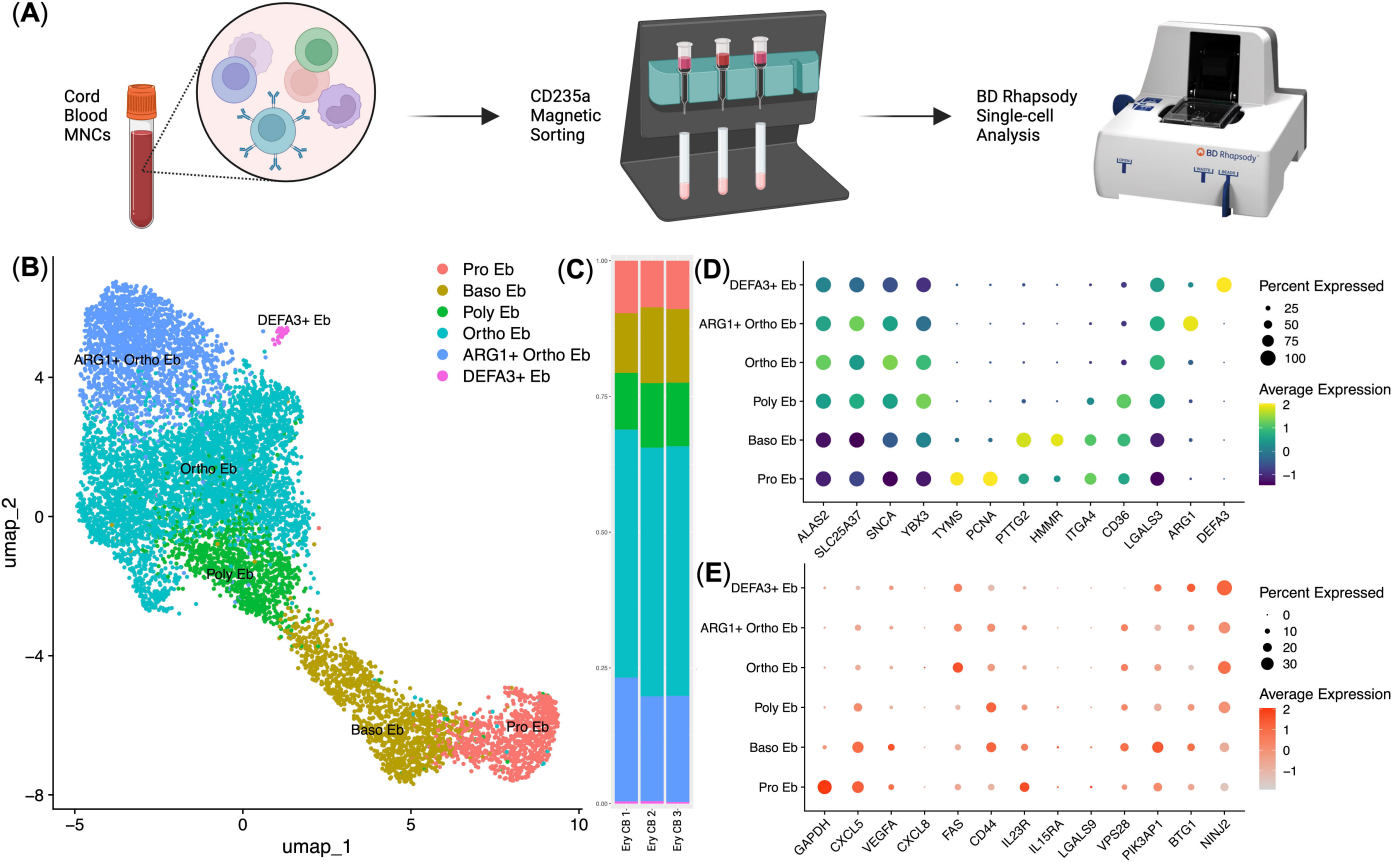
Immunotranscriptomic analysis of single human cord blood erythroid cells (*n* = 3, 9.615 single cells). **(A)** Overview of the experiment; **(B)** UMAP plot of the erythroid cell clusters; **(C)** Stacked bar plot of the erythroid cell clusters, clusters are colored in accordance with the subfigure **(B)**; **(D)** erythroid cell cluster-defining gene expression dot plot, gene expression values are Z-score-transformed, dot size represents the percentage of cell positive for the marker; **(E)** Dot plot of the genes with minor expression in human cord blood erythroid cells, gene expression values are Z-score-transformed, dot size represents the percentage of cell positive for the marker.

To investigate the human analog of murine E19.5 Ter-119^+^ erythroid cells, we performed scRNA-seq of cord blood erythroid cells obtained at birth from the placenta using the BD Rhapsody platform (**Figure 6A**). Our analysis identified distinct erythroid cell clusters corresponding to different maturation stages, including proerythroblasts (Pro Eb), basophilic erythroblasts (Baso Eb), polychromatic erythroblasts (Poly Eb), orthochromatic erythroblasts (Ortho Eb) using key markers of human erythroid cells. We used expression of *ALAS2* and *SLC25A37* genes to confirm erythroid identity, and *ALAS2, SLC25A37, TYMS, PCNA, ITGA4, HMMR, CD36*, and *PTTG2* genes to establish erythroid cell differentiation status.

We found that the immunosuppressive immunometabolic gene *ARG1* and antimicrobial immunity gene *DEFA3* defined two specialized subpopulations: *ARG1* ^+^ Ortho Eb and *DEFA3* ^+^ Eb (**Figures 6B, D**). *ARG1* ^+^ Ortho Eb and *DEFA3* ^+^ Eb on average comprised 20% and 2% of human cord blood erythroid cells, respectively (**Figure 6C**). Furthermore, cytokine gene expression analysis revealed distinct patterns across erythroid cell stages of differentiation, with CXCL5 and VEGFA expression highest in early progenitors and decreasing in more mature populations (**Figure 6E**). We also compared gene expression between human cord blood erythroid cells and mouse placental erythroid cells and found that these cells had two distinct gene expression profiles with little overlap between the expression profiles of their immunoregulatory molecules: they shared gene expression of the *GAPDH, S100A9, IL1B, STAT1, STAT3, CD74, CCL4, CXCR4, TGFB3*, and *CLEC4E* genes, human cord blood erythroid cells had unique gene expression of proinflammatory cytokine *IL18*, and mouse placental erythroid cells had unique gene expression of *Cxcl1, Cxcl12, Ccl2, Ccl3*, and *Ccl9* chemokines (**Supplementary Figures 7**, **8**).

## 4 Discussion

In this study, we conducted a multi-omics analysis that included bulk and single-cell transcriptomics and proteomics, spatial transcriptomics, and bulk secretomic profiling of murine placental and human cord blood erythroid cells to investigate their immunoregulatory properties. Our findings reveal that erythroid cells represent a previously underappreciated immunoregulatory cell population in the murine placenta, displaying gene expression of both immunosuppressive and antimicrobial genes.

Spatial transcriptomics and flow cytometry analysis of murine placentas and placental mononuclear cells confirmed that erythroid cells constitute a substantial proportion of murine placental immunoregulatory cells, substantially outnumbering conventional immune cell populations. These observed differences in immune cell composition between human and mouse placentas could be potentially explained by gestational duration and immune demands. Human pregnancy takes 9 months and requires long-lasting and complicated immune control, whereas the average mouse pregnancy lasts only 21 days (47, 48). In this context, non-specialized erythroid cells might provide sufficient protection in mice, due to their molecular toolkit of immunoregulatory molecules. Our findings indicated that murine placental erythroid cells exhibited high expression of immune-modulatory cytokines and chemokines, including *Tgfb1, Tgfb3, Cxcl1, Cxcl12, Ccl2, Ccl3*, *Ccl4*, and *Ccl9*, which are known to mediate immune suppression and maintain tissue homeostasis. Notably, murine placental erythroid cells expressed high levels of PD-L1 (*Cd274* gene) and genes involved in MHC class II antigen presentation. This raises the hypothesis that these cells may contribute to immune tolerance by presenting fetal antigens to T-cells with immunosuppressive co-stimuli, thus thwarting immune rejection (26), which may also explain why erythroid cells are particularly enriched in the placenta during pregnancy.

We also observed a temporal shift in both cell percentage and the functional profile of murine placental erythroid cells. At E12.5, erythroid cells exhibited nearly identical transcriptomic profiles. However, by E19.5, they showed a 2-fold decrease in abundance and an upregulation of inflammatory and antimicrobial genes such as *Il1a, Il1b, Clec4e, S100a8*, and *S100a9* in syngeneic pregnancy, and an upregulation of TGF-beta signaling pathway genes in semi-allogeneic pregnancy. These findings suggest that placental erythroid cells may dynamically regulate immune responses based on the presence or absence of paternal antigenic stimuli – promoting antibacterial immune response in the absence of such antigenic conflict in syngeneic pregnancy where microbial antigens are the main potential danger (which as we showed is possible *in vitro*), or promoting immune tolerance to eliminate such conflict in semi-allogeneic pregnancy. The decrease in erythroid cell abundance at E19.5 might be required to reduce the overall immunosuppressive microenvironment to induce inflammatory stimuli that could help the birthing process (24, 26, 27). We have previously described the antibacterial properties of human and mouse erythroid cells (37), suggesting that antibacterial activity may be a conserved mechanism among such cells.

The comparison of murine placental erythroid cells with classical M1 and M2 macrophages provided further insights into their immunoregulatory nature. Our analysis revealed that murine placental erythroid cells have overlapping expression of multiple immunoregulatory genes, such as MHC class II machinery genes, at comparable levels with both macrophage subsets. This suggests that murine nucleated erythroid cells can be considered a significant and effective immunoregulatory cell population.

The differences in cytokine production profiles between syngeneic and semi-allogeneic pregnancy erythroid cells indicate that antigenic disparity might influence erythroid cell function. We propose that recognition of non-self-antigens alters erythroid cell cytokine production via indirect signaling, either through maternal immune interactions or placental stromal cues, leading to enhanced production of TGF-β1 in semi-allogeneic settings.

Our analysis of murine placental erythroid cell counterparts that are human placenta-derived cord blood erythroid cells further supports the immunosuppressive role of erythroid cells in fetal development, regardless of species. We identified a specialized *ARG1^+^* orthochromatic erythroblast subpopulation, which may contribute to immune suppression by modulating arginine metabolism in immune cells – a critical pathway for the function of activated antigen-specific immune cells (49–51). Additionally, human erythroid cells express the gene for the antimicrobial peptide Alpha 3 Defensin (*DEFA3* gene), which reinforces the idea that erythroid cells in both humans and mice play a role not only in regulating immune responses but also in defending against potential microbial infections (52, 53). Interestingly, while murine placental erythroid cells exhibited MHC class II expression, human erythroid cells did not, suggesting species-specific differences in erythroid-mediated immune modulation. The expression of the *VEGFA* gene also suggests that human cord blood erythroid cells could support the development of the blood vessels (54, 55) in both the fetus and placenta, potentially facilitating enhanced blood flow to supply the fetus with the required metabolites. Overall, human cord blood erythroid cells were identical in the spectrum of expressed immunoregulatory molecules to their adult bone marrow and fetal liver counterparts, as they all expressed the immunoregulatory molecules *ARG1, CXCL5, IL1B, IL18*, and *VEGFA* (37, 42, 43).

It is worth noting that the erythroid cells of the mouse placenta and human cord blood turned out to be slightly different populations in terms of the spectrum of expressed immunoregulatory molecules. Yet, despite the differences in the spectrum of expressed molecules, there were also common genes associated with the antimicrobial response (*CCL4*, *CXCR4, CLEC4E, STAT1, STAT3, IL1B*, and *S100A9*). A summary of common and different features of gene expression in human and mouse erythroid cells at fetal development stages is presented in **Table 1**.

**Table 1:** Similarities and differences between human and mouse fetal-development-stage erythroid cell immunoregulatory gene expression.

Erythroid cells	Cytokines	Chemokines	Immuno- suppression	Antimicrobial immunity	MHC class II expression
Mouse	*Il1a, Il1b*	*Cxcl1, Cxcl12, Ccl2, Ccl3, Ccl4, Ccl9, Mif*	*Cd274* (PD-L1)*, Tgfb1, Tgfb3*	*Clec4e, S100a8*, *S100a9*	+
Human	*IL1B, IL18*, *VEGFA*	*CCL4, CXCL5*	*ARG1, TGFB3*	*CLEC4E, DEFA3, S100A9*	–

A limitation of this study is that cell viability was assessed solely by trypan blue exclusion, without the use of more sensitive fluorescent dyes such as 7-AAD or propidium iodide. As a result, minor membrane damage or early apoptotic events may have gone undetected, potentially leading to an overestimation of viable cells.

In conclusion, our findings highlight placental erythroid cells as potent players in shaping the immune landscape of murine pregnancy. Their immunosuppressive properties are likely to contribute to maternal-fetal tolerance by preventing excessive immune activation and offering protection against microbial threats. Future studies are needed to validate the proposed mechanisms by which erythroid cells modulate immune responses and to explore their potential clinical implications in pregnancy-related complications and immune disorders.

## Data Availability

The datasets presented in this study can be found in online repositories. The names of the repository/repositories and accession number(s) can be found below: https://www.ncbi.nlm.nih.gov/search/all/?term=GSE289175, GSE289175 https://doi.org/10.5281/zenodo.14794860.
