# Supplementary material for: Multi-omic analysis identifies erythroid cells as the major population in mouse placentas expressing genes for antigen presentation in MHC class II, chemokines, and antibacterial immune response
Source: Front Immunol. 2025 Sep 22;16:1644983. doi: 10.3389/fimmu.2025.1644983 (PMC12497581; doi:10.3389/fimmu.2025.1644983)
Supplement: Supplementary file 1 [file DataSheet1.pdf]

Supplementary Material

Spatial transcriptomic analysis of murine placentas

Preparation of 10X Genomics Visium Spatial Transcriptomics Libraries

We fixed placental sections (*n* = 3 for each type of pregnancy at E12.5) in 30 ml of 100% methanol at -20°C for 30 min and dehydrated them with 1 ml of 100% isopropanol for 5 min. We then embedded the dried sections in cassettes to select the section region for preparing the NGS library of the spatial transcriptome. We added a pre-hybridization buffer to the cassette-embedded sections and then removed it after 15 minutes of incubation.

Next, we added molecular probes that bind mouse gene transcripts within the section and hybridized the cassette-embedded sections with the added molecular probes at 50°C for 20 h using a Bio-Rad C1000 thermal cycler, a Bio-Rad 96-DeepWell thermal cycler adapter (Bio-Rad, Hercules, USA), and a 10X Genomics Visium cassette adapter (10X Genomics, Pleasanton, USA). After incubation, we removed the reaction mixture with probes and washed the cassette cavities with sections three times with 150 μl of post-hybridization buffer. We then washed them once with 150 μl of 2x SCC. Next, we ligated the probes at 37°C for 1 h and performed post-ligation washes.

We removed the sections from the cassettes, stained them with 250 µl of 0.05% aqueous Eosin-Y for 1 min, washed off the Eosin-Y, and transferred them to the 10X Genomics Visium CytAssist apparatus (10X Genomics, Pleasanton, USA) for tissue micrographing, tissue lysis, and probe transfer to a glass chip for spatial gene expression studies. To perform tissue lysis and probe transfer, we mounted two slides containing placenta sections with fetuses in the 10X Genomics Visium CytAssist against the gene expression capture chip so that the capture areas coincided with the regions of interest in the tissues. We then performed micrographing and tissue lysis at 37°C for 30 min using a tissue removal enzyme via the Visium CytAssist apparatus. After tissue lysis, we washed the glass chip containing the transferred ligated probes three times with 1 ml of 2x SCC.

We mounted the washed gene expression glass chip in a 10X Genomics Visium Gene Expression Assay Cassette and performed reverse transcription at 45°C for 15 minutes. After completing reverse transcription, we removed the reaction mixture and washed the cassette cavities with 2x SCC. We then added 0.08 M KOH to each cassette cavity and incubated for 10 min to dissociate cDNA from the glass-chip probes. We

transferred the dissociated cDNA to a new 1.5 ml tube and neutralized the reaction mixture with 12 µl of 1 M Tris-HCl pH 8.0.

We then performed PCR amplification of the obtained cDNA, followed by the purification of the resulting PCR mixture using SPRIselect beads. For final library preparation, we performed additional PCR amplification with Illumina-compatible primers, purified the final libraries using SPRIselect beads, and pooled them in proportions corresponding to the number of tissue-occupied spatial spots. We sequenced the libraries on an Illumina NovaSeq 6000 using an SP flow cell (700 million paired reads, a minimum of 35000 reads per spot, I5 = 10, I7 = 10, R1 = 31, R2 = 51).

Processing raw data from 10X Genomics Visium spatial transcriptomics libraries

We processed raw FASTQ R1 and FASTQ R2 reads and micrographs for each section using the 10X Genomics Space Ranger bioinformatics pipeline on Ubuntu Linux 20. Space Ranger automatically identified spatial points occupied by tissue, aligned reads to the mouse reference transcriptome, and identified transcript abundance and localization using UMI data and spatial barcodes. Space Ranger pipeline then generated a transcript abundance matrix, saved it as a "cloupe" file for Loupe Browser software, and exported gene x spot data as an h5 file and in the market exchange format and a PNG image for further bioinformatics analysis. We observed >97% sequencing saturation for all samples.

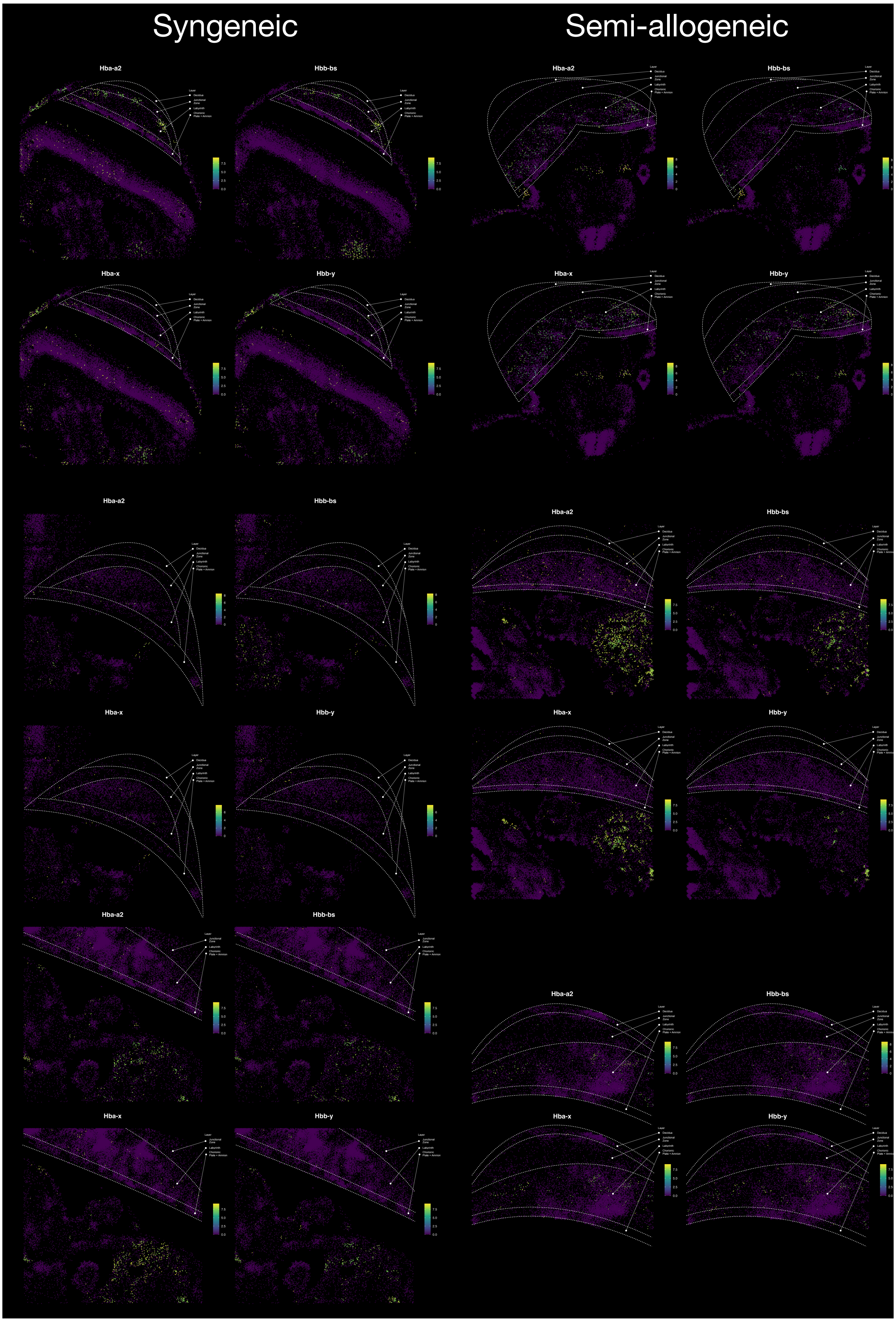

Supplementary Figure 1. Erythroid cell lineage normal and fetal hemoglobin chain gene expression for each type of pregnancy at E12.5 (*n* = 3). Dashed lines indicate placental layers; cells outside the explicitly outlined placental layers belong to fetal and perifetal tissues.

Cell Type	Syngeneic E12.5				Semi-Allogeneic E12.5			
Other Placental	3.574	4.161	3.784	7.350	10.171	6.047	3.005	10.180
Other CD45+ Immune	0.727	5.292	1.872	4.247	8.191	3.883	3.890	9.077
CD4 T-cells	0.542	0.094	0.081	0.008	0.291	0.038	0.384	0.022
CD8 T-cells	6.505	1.916	1.077	0.954	6.012	5.132	6.326	0.536
Monocytes	1.210	1.243	0.586	0.799	5.885	2.509	3.365	0.197
Nucleated Erythroid Cells	40.164	40.407	40.311	36.302	32.170	41.065	40.947	33.178
Nucleated Reticulocytes	47.278	46.887	52.289	50.340	37.280	41.326	42.083	46.810
Sum Erythroid cells	87.442	87.294	92.600	86.642	69.450	82.391	83.030	79.988
**Cell Type**	**Syngeneic E19.5**				**Semi-Allogeneic E19.5**			
Other Placental	59.496	53.040	41.088	50.911	48.112	40.864	59.958	46.326
Other CD45+ Immune	2.444	1.010	2.984	4.277	3.609	7.801	1.475	6.743
CD4 T-cells	0.036	0.031	0.057	0.046	0.056	0.068	0.013	0.008
CD8 T-cells	1.296	1.730	1.662	2.298	6.125	3.505	0.816	1.678
Monocytes	0.203	0.209	0.915	0.219	0.913	0.498	0.072	0.055
Nucleated Erythroid Cells	22.297	25.749	35.417	29.160	28.047	33.074	23.922	30.417
Nucleated Reticulocytes	14.228	18.231	17.877	13.089	13.138	14.190	13.744	14.773
Sum Erythroid cells	36.525	43.980	53.294	42.249	41.185	47.264	37.666	45.190

Supplementary Table 1. Placental mononuclear cell population percentages (*n* = 4).

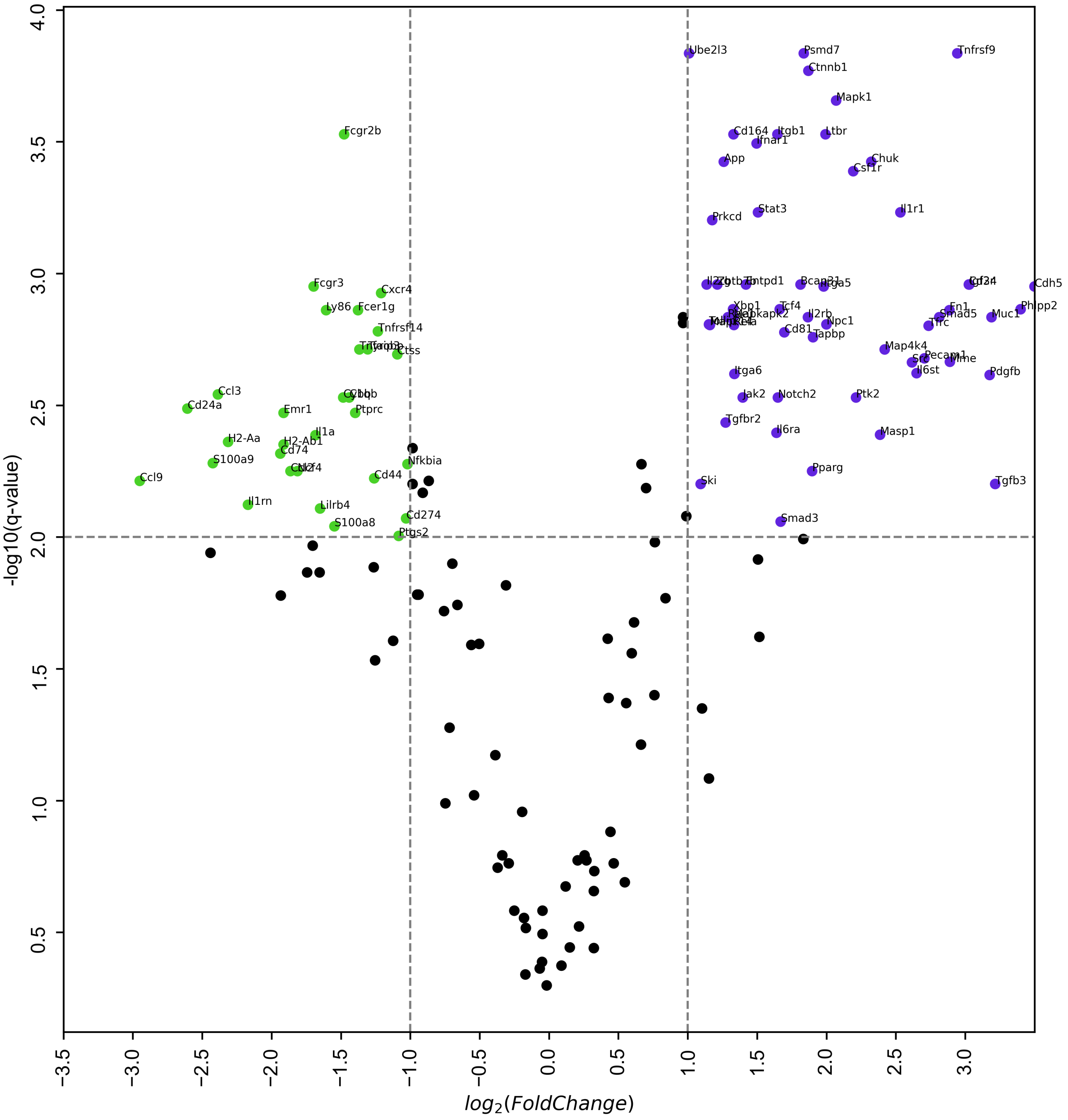

Supplementary Figure 2. Volcano plot of differentially expressed genes in semi-allogeneic E19.5 mouse placenta erythroid cells compared to syngeneic E19.5 mouse placenta erythroid cells.

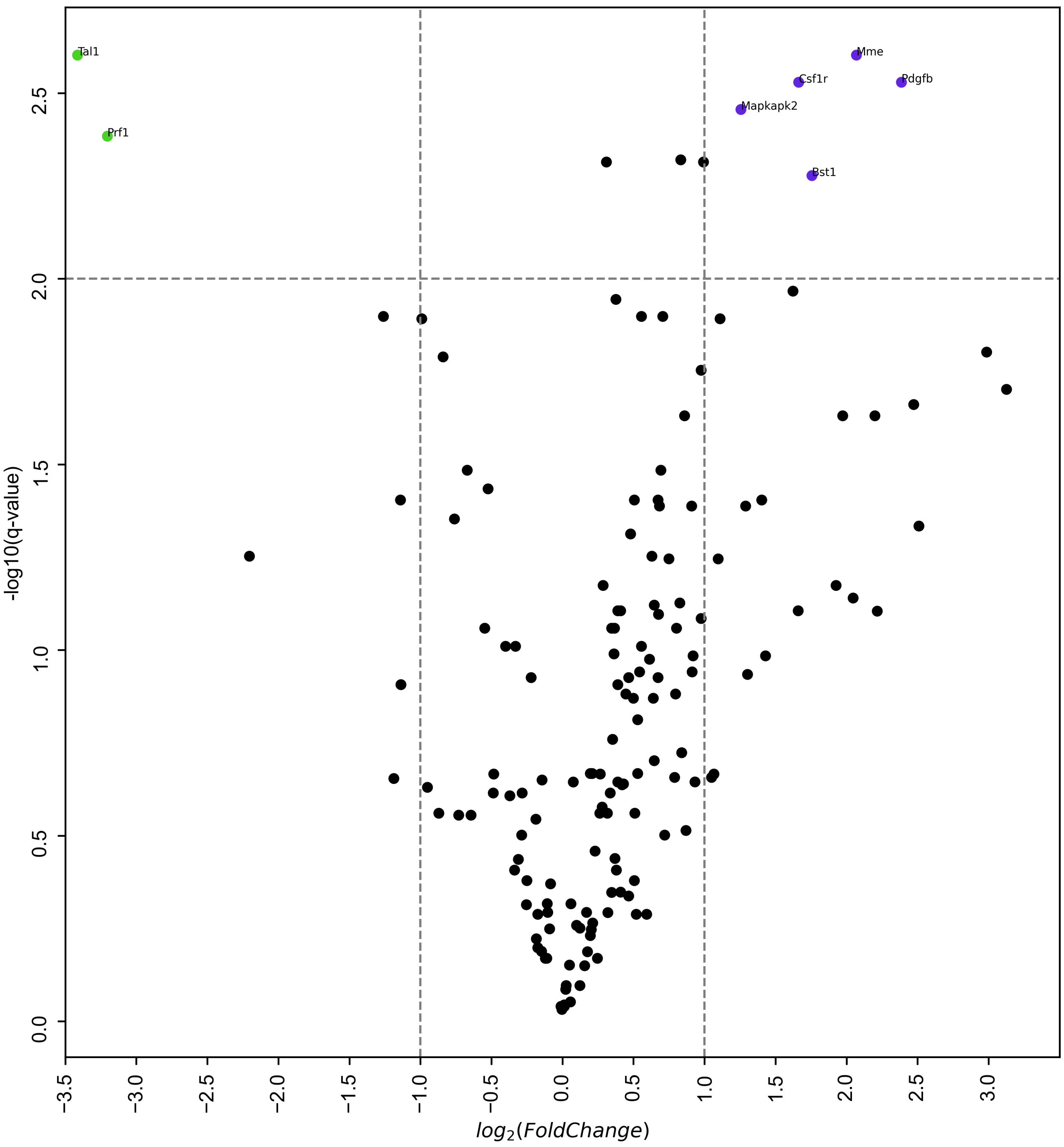

Supplementary Figure 3. Volcano plot of differentially expressed genes in semi-allogeneic E19.5 mouse placenta erythroid cells compared to semi-allogeneic E12.5 mouse placenta erythroid cells.

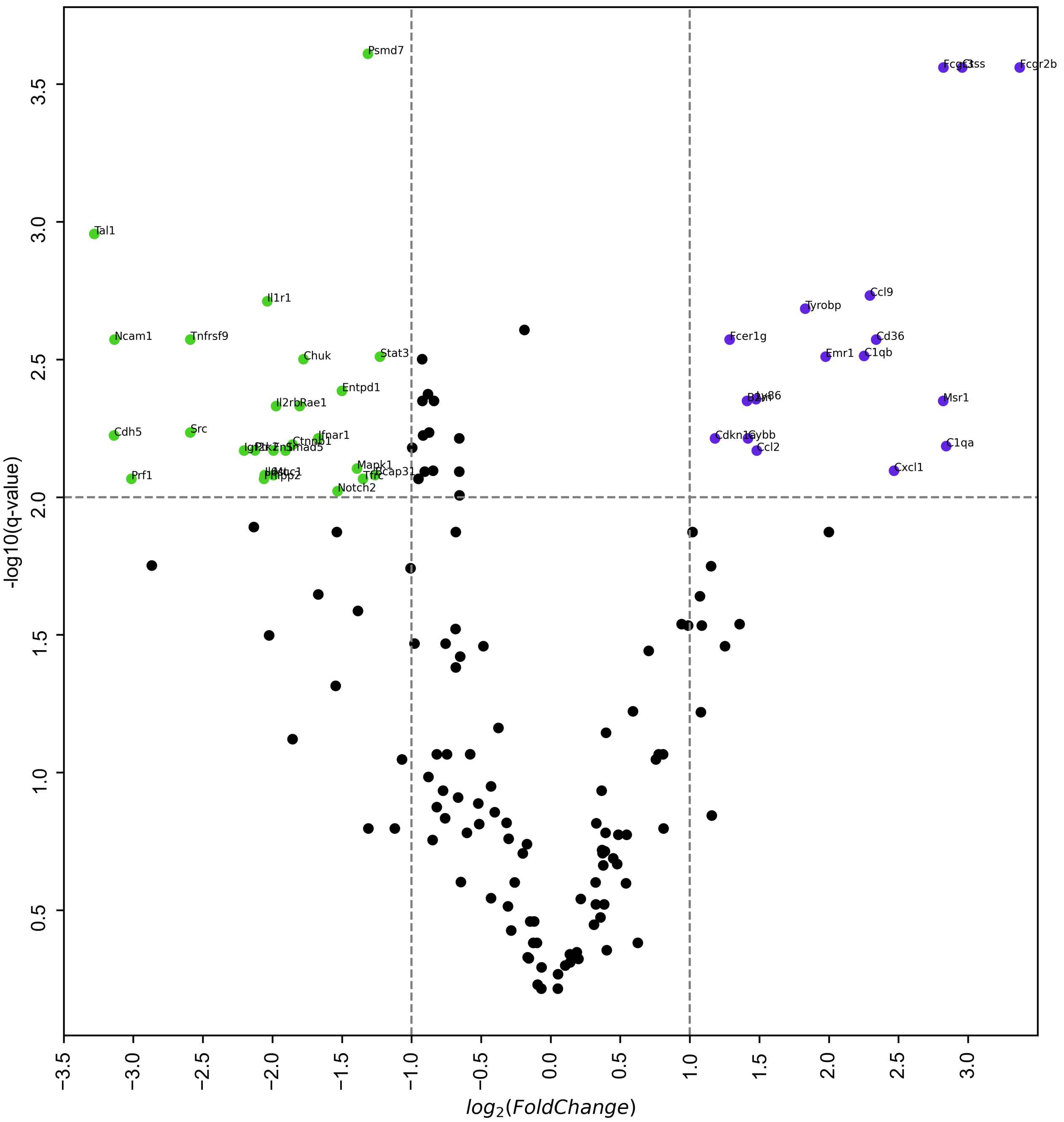

Supplementary Figure 4. Volcano plot of differentially expressed genes in syngeneic E19.5 mouse placenta erythroid cells compared to syngeneic E12.5 mouse placenta erythroid cells.

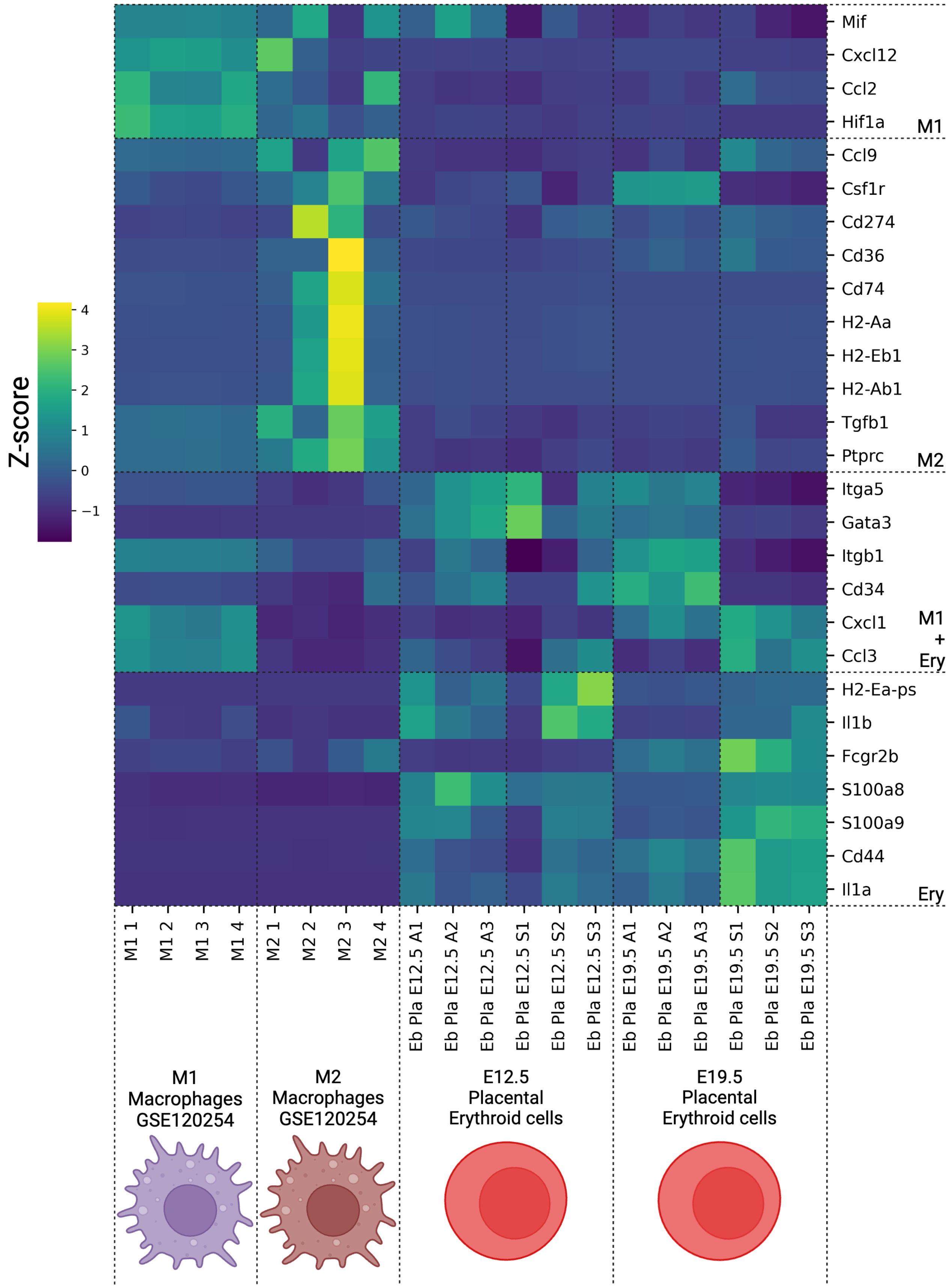

Supplementary Figure 5. Heat map of the common genes between the murine placental Erythroid cells at E12.5 and E19.5 from syngeneic and semi-allogeneic pregnancies and M1 and M2 macrophages.

Sample	IL-34	CXCL 12	IL-6	GM-CSF	TGF-β1	SCF	TPO	IL-5	IL-15	LIF	EPO	IL-3	M-CSF
**Pla E12.5 A Eb 1**	11233	10225	2688	1110	630	703	1735	194	130	183	0	0	0
**Pla E12.5 A Eb 2**	8178	15700	4930	528	1125	343	530	125	105	120	0	0	0
**Pla E12.5 A Eb 3**	9510	11325	1590	1440	1055	405	765	68	88	98	0	0	0
**Pla E12.5 A Eb 4**	9528	11343	2705	1128	1073	423	783	142	123	138	0	0	0
**Pla E12.5 S Eb 1**	12435	4199	78	6973	0	178	2783	1524	538	0	0	0	0
**Pla E12.5 S Eb 2**	12595	2108	1117	3362	0	178	1153	617	229	40	0	0	0
**Pla E12.5 S Eb 3**	18300	4163	1103	1440	0	103	410	194	113	50	0	0	0
**Pla E12.5 S Eb 4**	23520	6325	2170	1673	0	433	268	133	38	70	0	0	0
**Pla E19.5 A Eb 1**	665	325	1338	583	125	110	48	0	0	335	0	0	0
**Pla E19.5 A Eb 2**	1275	2625	773	1110	142	165	143	11	5	20	0	0	0
**Pla E19.5 A Eb 3**	648	1485	1065	565	123	93	105	16	13	188	0	0	0
**Pla E19.5 A Eb 4**	665	1503	1083	583	140	110	123	29	23	205	0	0	0
**Pla E19.5 S Eb 1**	5293	2775	133	1163	0	150	155	56	38	95	0	0	0
**Pla E19.5 S Eb 2**	9510	750	150	1218	0	223	480	232	40	33	0	0	0
**Pla E19.5 S Eb 3**	5280	1475	150	48	0	243	225	81	13	28	0	0	0
**Pla E19.5 S Eb 4**	5293	1488	163	1175	0	235	238	94	50	45	0	0	0

Supplementary Table 2. LegendPlex cytokine concentration (pg/mL) profiles of placental (Pla) Ter-119$^+$ Erythroid cell (Eb)-derived conditional media at E12.5 and E19.5 in both syngeneic (S) and semi-allogeneic pregnancies (A) (n = 4).

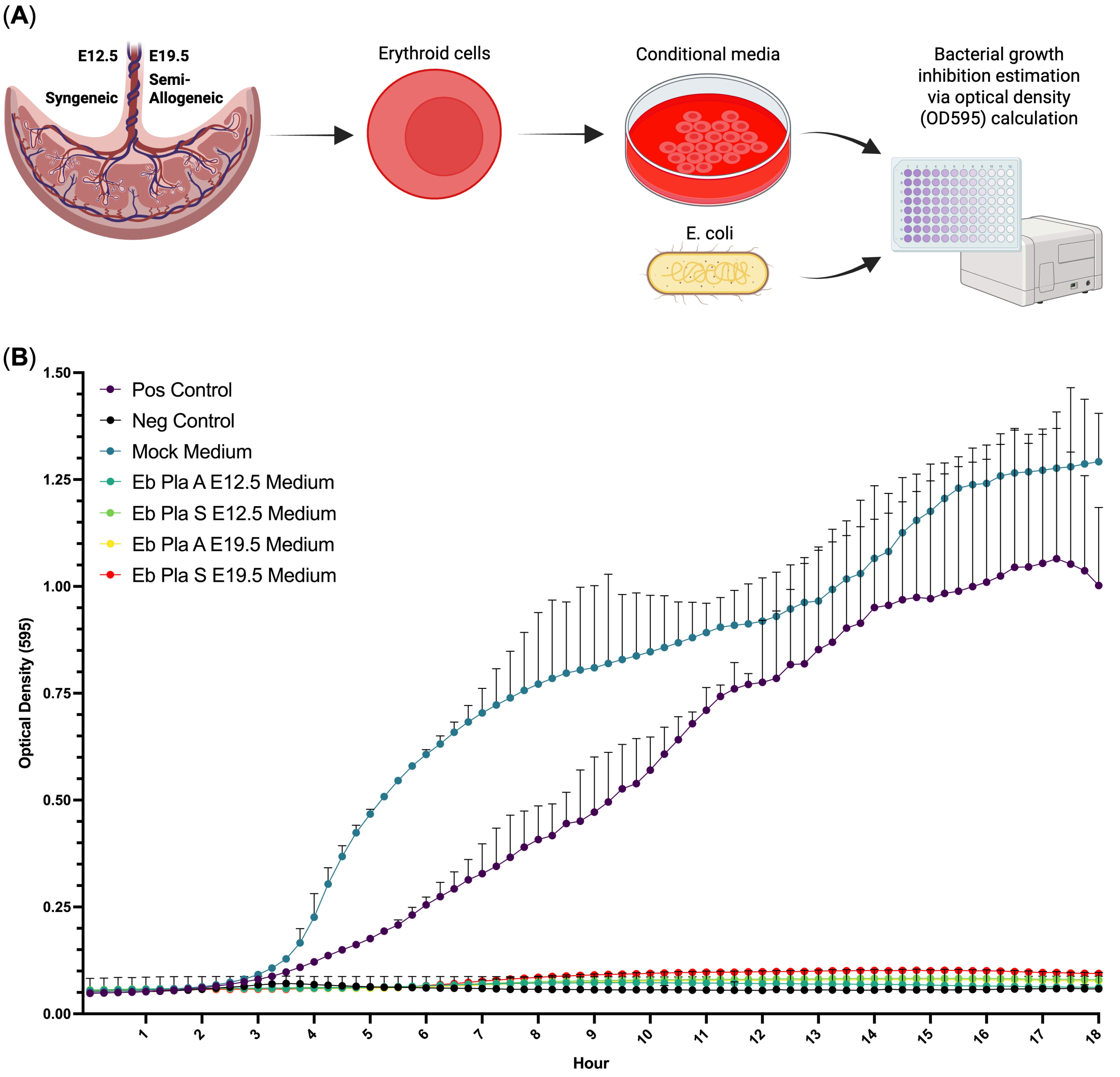

Supplementary Figure 6. Bacterial growth inhibition effect of the murine placental (Pla) Erythroid cell (Eb) conditional media from syngeneic (S) and semi-allogeneic (A) pregnancies at E12.5 and E19.5 (*n* = 6). (**A**) Overview of the experiment, (**B**) Dot plot of incubation time versus the detected median OD595 values.

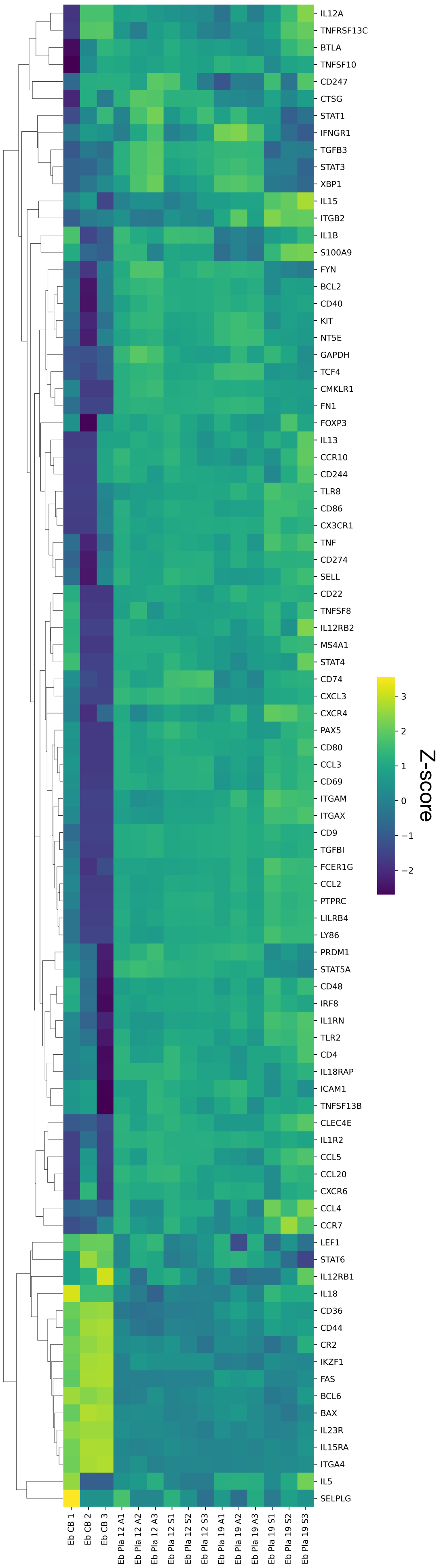

Supplementary Figure 7. Heatmap of the genes with shared expression between human cord blood (CB) and murine placenta (Pla) erythroid cells (Eb).

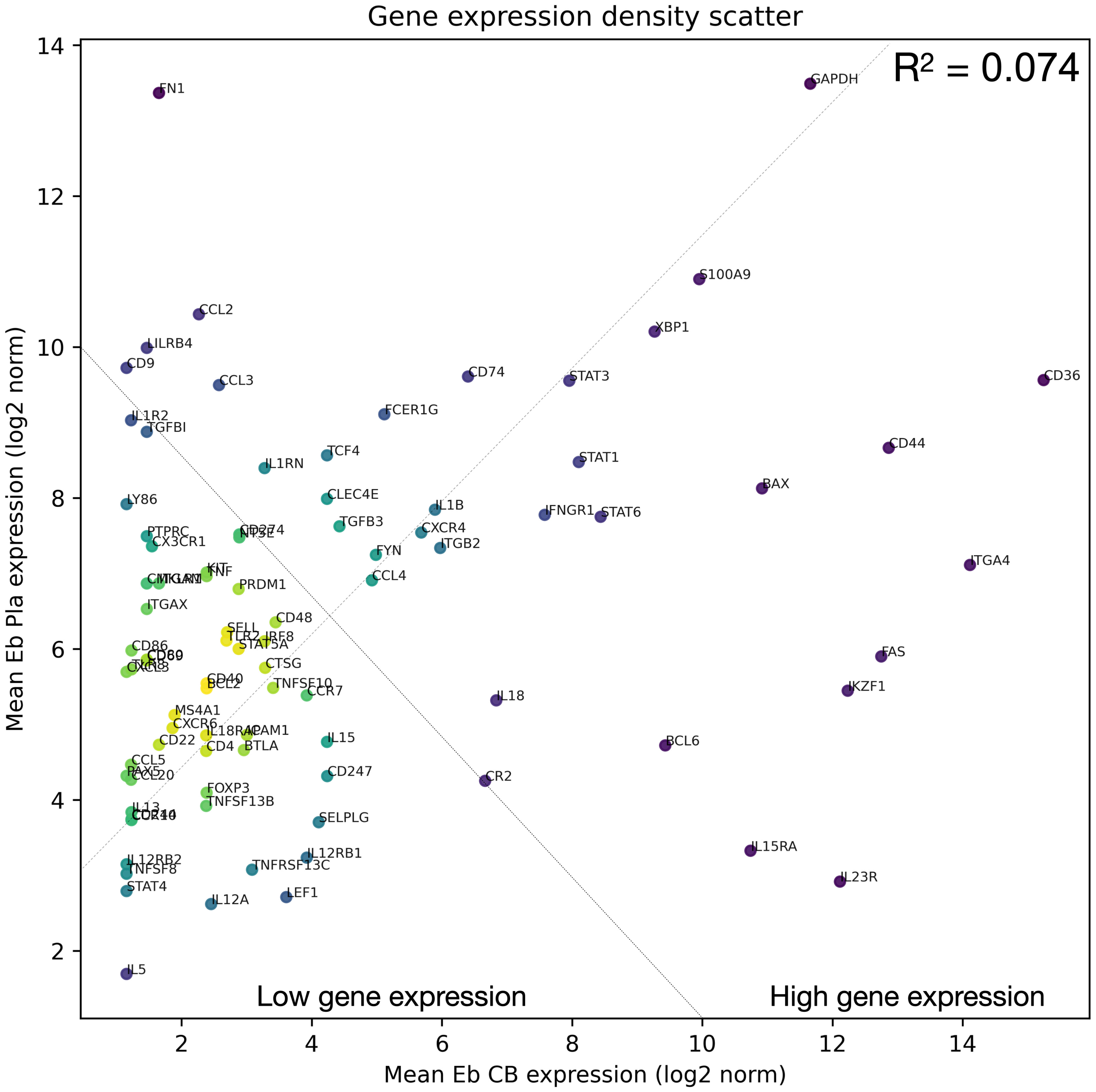

Supplementary Figure 8. Density plot of the differences and similarities in the gene expression between human cord blood (CB) and murine placenta (Pla) erythroid cells (Eb).
